# Harmonizing Medicine and Surgery in the Pursuit of Boolean Remission: A Rheumatological Magnum Opus

**DOI:** 10.7759/cureus.48205

**Published:** 2023-11-03

**Authors:** Abdur Rehman, Jinal Choudhari, Abdullah Shehryar, Maryam Affaf, Hareem Ata, Wajiha Batool, Bilal Khan, Iti Mehra, Rayan W Gasim, Quratulain Fatima Masood, Nabila N Anika, Shehryar Rehman

**Affiliations:** 1 Surgery, Mayo Hospital, Lahore, PAK; 2 Division of Research & Academic Affairs, Larkin Community Hospital, Miami, USA; 3 Internal Medicine, Allama Iqbal Medical College, Lahore, PAK; 4 Internal Medicine, Women’s Medical & Dental College, Abbotabad, PAK; 5 Internal Medicine, National University of Science and Technology, Rawalpindi, PAK; 6 Internal Medicine, Army Medical College, Rawalpindi, PAK; 7 General Surgery, Jinnah Postgraduate Medical Centre, Karachi, PAK; 8 Internal Medicine, Emilio Aguinaldo College, Manila, PHL; 9 Internal Medicine, University of Khartoum, Khartoum, SDN; 10 Surgery, National University of Science and Technology, Rawalpindi, PAK; 11 Surgery, Holy Family Red Crescent Medical College and Hospital, Dhaka, BGD; 12 Internal Medicine, Al-Assad University Hospital, Damascus, SYR

**Keywords:** treat-to-target, surgical interventions, pharmacological treatments, rheumatic diseases, rheumatoid arthritis (ra), boolean remission

## Abstract

Rheumatic diseases encompass a diverse group of musculoskeletal conditions that often lead to inflammation, pain, and significant limitations in patients' lives. While traditional treatment approaches have primarily centered on medications to control symptoms, recent developments have introduced the concept of Boolean remission. Boolean remission offers a comprehensive evaluation of disease activity by considering clinical, biochemical, and patient-reported outcomes. This narrative review explores the multifaceted landscape of Boolean remission in the context of rheumatic diseases, with a focus on rheumatoid arthritis (RA), as it remains a substantial clinical challenge. The review outlines the definition, criteria, historical context, and development of Boolean remission, shedding light on its emergence as a more patient-centered and stringent treatment goal. The role of pharmacological interventions, including immunomodulators and biologics, in achieving Boolean remission is discussed, emphasizing the significance of treatment protocols that encompass regular monitoring, medication adjustment, shared decision-making, and patient education. Surgical interventions, such as joint replacements and synovectomies, complement medication-based strategies when joint damage becomes severe, with adherence to surgical protocols ensuring sustained Boolean remission. The integration of medicine and surgery through integrated care models and interdisciplinary teams is examined as a critical aspect of optimizing patient outcomes. Boolean remission's broader impact on healthcare policies and clinical trial endpoints is explored, underscoring its growing significance in rheumatic disease management. The review concludes by looking toward the future, where emerging technologies, biomarkers, and personalized medicine approaches hold promise in refining Boolean remission criteria and making it a more attainable and impactful treatment goal. Policy implications suggest the integration of Boolean remission into healthcare quality metrics, incentivizing healthcare providers to prioritize this rigorous standard of care. Boolean remission represents a pivotal shift in the holistic and patient-centered management of rheumatic diseases, offering hope for improved patient outcomes and enhanced quality of life in this challenging clinical landscape.

## Introduction and background

Rheumatic diseases encompass a varied group of conditions characterized by inflammation, pain, and limitations in the musculoskeletal system. These can range from localized joint disorders, such as osteoarthritis, to systemic autoimmune diseases, such as rheumatoid arthritis (RA) and lupus. These conditions not only affect physical health but also significantly influence patients' quality of life, often leading to psychosocial comorbidities [1]. Traditionally, the treatment of rheumatic diseases was medication-focused, particularly on immunomodulating agents to control inflammation. However, in more severe cases that do not respond well to medications, surgical interventions, such as joint replacements or synovectomies, become necessary. The integration of medicine and surgery offers a more holistic treatment paradigm, potentially more effective than either approach in isolation [2].

In recent years, the rheumatology community has witnessed a paradigm shift with the introduction of the concept of Boolean remission in the management of RA. This innovative approach has redefined how clinicians and researchers perceive and measure remission, moving beyond the limitations of traditional definitions that relied primarily on either biochemical markers or subjective patient reports. Boolean remission offers a multi-dimensional perspective, incorporating a comprehensive set of measures that include clinical signs, biochemical indicators, and patient-reported outcomes to provide a more holistic assessment of disease activity [3]. This nuanced approach allows for a more accurate and complete understanding of each patient's disease state, thereby facilitating more targeted treatment strategies. Despite the promise of this robust diagnostic tool, achieving a state of sustained remission remains a complex challenge. Various studies have shown that even when remission is initially achieved, it may not be durable in the long term, necessitating continued monitoring and treatment adjustments [4]. The elusive nature of sustained remission has driven the need for continuous research and clinical trials to better understand the factors that contribute to the persistence of disease activity. The primary aim of this narrative review is to investigate the harmonization of medical and surgical approaches for achieving Boolean remission in rheumatic diseases, especially RA.

This review will examine the latest evidence, discuss challenges, and look toward future directions in achieving effective and sustained remission. Despite advances in treatments, achieving true remission remains a significant hurdle for many patients [5].

## Review

What is Boolean remission?

Boolean remission has emerged as a transformative concept in the field of rheumatology, especially in the management of RA. This groundbreaking paradigm goes beyond the limitations of traditional definitions of remission, which often focus exclusively on singular aspects, such as clinical signs or biochemical markers. Boolean remission embraces a more multi-faceted approach, amalgamating a variety of measures to offer a fuller understanding of a patient's condition. It encompasses clinical indicators, such as the tender joint count (TJC) and swollen joint count (SJC), alongside biochemical markers, such as C-reactive protein (CRP) levels. Moreover, it incorporates patient-reported outcomes, such as the Patient Global Assessment (PGA) and Health Assessment Questionnaire (HAQ), to gauge the patient's perception of their well-being and functional ability [6]. By integrating these multiple dimensions, Boolean remission provides a more rigorous and holistic framework for assessing the true status of a patient's disease activity. This allows clinicians and researchers alike to capture a more accurate snapshot of disease activity, thereby facilitating more effective treatment plans and better patient outcomes.

Criteria for Boolean Remission

Attaining Boolean remission necessitates fulfilling a well-defined set of criteria that serve as benchmarks for disease control and patient well-being [6]. These criteria are designed to offer a holistic picture of the patient's condition by integrating multiple dimensions, namely, clinical signs, biochemical markers, and patient-reported outcomes. Below are the key criteria:

TJC: Clinicians assess the number of tender joints through physical examination. To meet the criteria for Boolean remission, patients should not have more than one tender joint, reflecting reduced localized inflammation and pain.

SJC: This criterion involves a clinical evaluation of the number of swollen joints, which should similarly be no more than one. A low SJC indicates effective control of joint inflammation, a hallmark of rheumatic diseases.

CRP level: CRP is a biochemical marker of systemic inflammation. For Boolean remission, CRP levels should be within the normal range, usually less than or equal to 1 mg/dL, denoting that the underlying inflammatory process is under control [7].

PGA: This patient-reported measure captures the individual's perception of their disease activity. A low score on a visual analog scale (VAS) often signifies that the patient feels that their condition is well managed.

Pain on the VAS: Pain is a significant factor affecting the quality of life in rheumatic diseases. A minimal score on the VAS suggests that the patient is experiencing low levels of pain, an indicator of effective symptom management.

Health assessment questionnaire (HAQ): The HAQ score measures functional ability, including daily activities and mobility. A low score indicates that the patient's functional capacity is largely intact, enabling them to lead a more normal life [8].

Meeting all of these criteria collectively indicates that the patient has achieved Boolean remission, meaning that their disease activity is either significantly reduced or effectively nullified.

The concept of Boolean remission emerged as a response to the limitations of traditional definitions of remission in rheumatic diseases. Historically, remission was primarily assessed based on a reduction in disease activity scores or the absence of specific clinical symptoms. However, it became evident that some patients who met these criteria still experienced a significant impact on their daily lives.

The development of Boolean remission criteria aimed to provide a more comprehensive and patient-centered approach to evaluating disease activity. This approach considers not only clinical and biochemical markers but also the patient's perspective, encompassing their pain, functional ability, and overall well-being.

**Figure 1:**
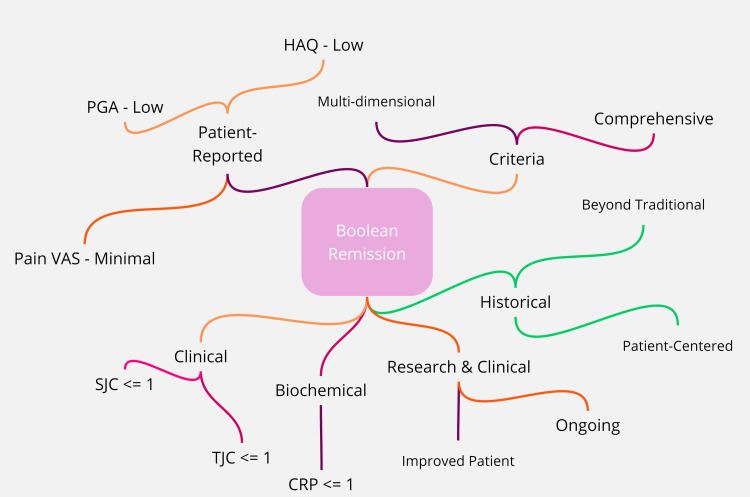
Boolean remission in rheumatoid arthritis: a comprehensive mindmap *Clinical indicators*: TJC (tender joint count) <= 1: indicates the number of tender joints through physical examination; SJC (swollen joint count) <= 1: represents the number of swollen joints, reflecting control of joint inflammation *Biochemical markers*: CRP (C-reactive protein) <= 1 mg/dL: a marker of systemic inflammation, with levels within the normal range suggesting controlled inflammation *Patient-reported outcomes*: PGA (Patient Global Assessment) - low score: a subjective measure of disease activity from the patient's perspective; pain VAS (visual analog scale) - minimal score: assesses the patient's pain level, with lower scores indicating effective pain management; HAQ (Health Assessment Questionnaire) - low score: measures functional ability, with lower scores indicating better functional capacity *Criteria integration*: multi-dimensional assessment: integration of various measures for a holistic view of the patient's condition; comprehensive evaluation: a thorough approach that encompasses clinical, biochemical, and patient-reported data *Historical context*: beyond traditional definitions: the evolution from singular measures of remission to a multifaceted approach; patient-centered approach: incorporation of patient-reported outcomes for a more personalized assessment *Research and clinical trials*: ongoing refinement: continuous research to refine the criteria for Boolean remission; improved patient outcomes: the ultimate goal of achieving Boolean remission reflects better disease control and quality of life for patients. The image is generated by the authors.

Boolean remission criteria have evolved, with ongoing research and clinical trials refining the definitions and thresholds. As a result, achieving Boolean remission has become an important goal in the management of rheumatic diseases, especially RA, as it reflects a higher level of disease control and improved patient outcomes [4]. Figure 1 gives a mindmap of Boolean remission in RA.

The role of medicine in achieving Boolean remission

Pharmacological Approaches

Pharmacological interventions play a central role in the pursuit of Boolean remission in rheumatic diseases. These approaches aim to suppress inflammation, reduce symptoms, and halt disease progression. Several classes of medications are commonly used:

Non-steroidal anti-inflammatory drugs (NSAIDs): NSAIDs provide relief from pain and inflammation, making them a valuable component of symptom management. However, they do not alter the underlying disease course and are typically used as adjuncts.

Disease-modifying anti-rheumatic drugs (DMARDs): DMARDs form the cornerstone of pharmacological treatment for rheumatic diseases. Conventional DMARDs, such as methotrexate, aim to slow down disease progression by modulating the immune system [5].

Biologic DMARDs: Biologics are a newer class of medications that specifically target components of the immune system responsible for inflammation. They have revolutionized the treatment of rheumatic diseases, including RA, by achieving better control of disease activity and improving the likelihood of Boolean remission [5,6].

Immunomodulators and Biologics

Immunomodulators are essential in the management of rheumatic diseases, as they act to regulate the overactive immune response responsible for tissue damage and inflammation. Methotrexate, a commonly used immunomodulator, has demonstrated effectiveness in achieving Boolean remission by reducing inflammation and disease activity [7].

Biologic DMARDs, including tumor necrosis factor (TNF) inhibitors, interleukin inhibitors, and B-cell depleting agents, have significantly expanded treatment options for achieving Boolean remission. These biologics target specific molecules involved in the inflammatory cascade, leading to reduced disease activity and improved outcomes. Studies have shown that the use of biologic DMARDs in combination with conventional DMARDs can increase the likelihood of achieving Boolean remission in rheumatic diseases [8]. The overview of pharmacological strategies for achieving Boolean remission in rheumatoid arthritis is given in Table 1.

**Table 1:** Pharmacological approaches and biologics in achieving Boolean remission

Category	Description	Examples
Pharmacological approaches	Interventions aimed at suppressing inflammation, reducing symptoms, and halting disease progression	
Non-steroidal anti-inflammatory drugs (NSAIDs)	Provide symptomatic relief from pain and inflammation but do not alter the disease course	Ibuprofen, naproxen
Disease-modifying anti-rheumatic drugs (DMARDs)	Slow down disease progression by modulating the immune system	Methotrexate, leflunomide
Immunomodulators	Regulate the overactive immune response to reduce tissue damage and inflammation	
Methotrexate	A commonly used immunomodulator that has demonstrated effectiveness in achieving Boolean remission	
Biologics	Target specific components of the immune system responsible for inflammation	
Tumor necrosis factor (TNF) Inhibitors	Target and neutralize TNF, a substance in the body that causes inflammation in the joints	Etanercept, infliximab
Interleukin inhibitors	Block interleukins, proteins that play a role in the immune response	Anakinra, canakinumab
B-cell depleting agents	Target B-cells, which are a part of the immune system and can contribute to inflammation in RA	Rituximab

Treatment Protocols

Attaining Boolean remission is a complex undertaking that requires a meticulously planned and executed treatment protocol. Rheumatologists commonly employ a "treat-to-target" strategy, setting explicit objectives for disease control, one of which is often achieving Boolean remission [9]. The following key components constitute the core of these treatment protocols:

Regular monitoring: The cornerstone of effective management is continuous surveillance of the patient's condition. This involves regularly scheduled evaluations that utilize a combination of clinical assessments, biochemical tests, and patient-reported measures. Such comprehensive monitoring allows clinicians to track the patient's progress rigorously and make timely interventions.

Adjustment of medications: Tailoring medication regimens is an ongoing process, informed by the patient's response to treatment and current disease activity. The goal is to optimize the control of inflammation and alleviate symptoms. This may involve the use of immunomodulators, biologics, or other anti-inflammatory medications, which are adjusted in dosage or type depending on the patient's needs and response.

Shared decision-making: Effective treatment is a collaborative effort that involves both the healthcare provider and the patient. Open channels of communication enable the patient to voice their preferences and concerns, which are considered when tailoring treatment plans. This shared decision-making process ensures that treatment objectives align with the patient's personal goals and lifestyle.

Patient education: A crucial yet often overlooked aspect of treatment is patient education. Knowledgeable patients are better equipped to manage their conditions and adhere to treatment regimens. Educational initiatives focus on explaining the nature of the disease, available treatment options, and the critical role of medication and lifestyle adherence in achieving successful management [9].

By meticulously adhering to these comprehensive treatment protocols, healthcare providers increase the likelihood of achieving Boolean remission, thereby improving both short-term and long-term patient outcomes.

The role of surgery in achieving Boolean remission

Types of Surgical Interventions

Surgery holds a pivotal role in the multifaceted management of rheumatic diseases, particularly when pharmacological treatments alone fall short of achieving Boolean remission. Several surgical interventions, each targeting specific issues associated with these conditions, can be employed to enhance patient outcomes:

Joint replacement surgery: Often considered a last resort when other treatments have failed, joint replacement surgery aims to alleviate severe joint damage that significantly compromises a patient's quality of life. The procedure involves the surgical removal of the damaged joint components, which are then replaced by artificial prosthetic parts. Common examples include hip and knee replacements. The primary benefits of joint replacement surgery are marked pain relief and a substantial improvement in joint function and mobility.

Synovectomy: In conditions like RA, the synovial membrane lining the joint capsule often becomes inflamed, contributing to ongoing joint deterioration. Synovectomy surgically removes this inflamed tissue, thereby reducing pain and potentially slowing down the progression of joint damage [10]. This procedure is particularly useful in cases where medication alone is insufficient to control synovial inflammation.

Tendon repair: Rheumatic diseases can cause significant wear and tear on tendons, leading in some cases to partial or complete rupture. When this occurs, surgical intervention may be required to repair the damaged tendons, either by stitching the torn parts together or by replacing them with grafts. This surgical repair aims to restore the tendon's function and alleviate pain, facilitating a return to normal activities [11].

Each of these surgical options serves a unique purpose and is considered based on individual patient needs, the severity of their condition, and their responsiveness to pharmacological treatments. Through these interventions, surgery complements medical therapies in offering a comprehensive approach to achieving Boolean remission and improving the quality of life for those afflicted by rheumatic diseases.

Surgical Protocols

Achieving Boolean remission through surgical interventions requires adherence to established surgical protocols:

Patient selection: Careful patient selection is essential to identifying individuals who are likely to benefit from surgery. Factors, such as disease severity, failed pharmacological management, and the patient's overall health, should be considered [12].

Multidisciplinary approach: Collaboration among rheumatologists, orthopedic surgeons, and other specialists is crucial to comprehensively assess the patient and determine the most appropriate surgical intervention [13].

Preoperative optimization: Patients should undergo thorough preoperative assessments and optimization of their medical conditions to minimize surgical risks.

Postoperative care: Following surgery, a well-structured rehabilitation program is essential to ensure optimal recovery and functional outcomes.

Long-term monitoring: Patients who undergo surgery for rheumatic diseases require long-term monitoring to assess the sustainability of Boolean remission and address any potential complications [14].

Harmonizing medicine and surgery

Integrated Care Models

Achieving Boolean remission often requires a coordinated approach that harmonizes both medical and surgical interventions. Integrated care models have emerged as a valuable strategy to ensure seamless collaboration between medical and surgical teams, resulting in improved patient outcomes [15].

Interdisciplinary Teams

Interdisciplinary teams, comprising rheumatologists, orthopedic surgeons, physical therapists, and other specialists, play a pivotal role in the harmonization of medicine and surgery. Their collaborative efforts aim to provide comprehensive care, optimize treatment plans, and enhance the likelihood of achieving Boolean remission [13].

Utility of Boolean remission in specific rheumatic diseases

Rheumatoid Arthritis

Boolean remission is of significant utility in RA management. It goes beyond traditional measures by incorporating clinical, biochemical, and patient-reported outcomes [4]. Achieving Boolean remission in RA has been associated with improved long-term outcomes, reduced joint damage, and enhanced quality of life [10].

Psoriatic Arthritis

In psoriatic arthritis (PsA), Boolean remission serves as a valuable treatment goal. This comprehensive remission definition accounts for skin involvement, joint symptoms, and inflammation markers, allowing for a more thorough assessment of disease control [11]. Attaining Boolean remission in PsA is linked to improved skin and joint outcomes, highlighting its utility.

Systemic Lupus Erythematosus

Boolean remission has also found utility in systemic lupus erythematosus (SLE) management. By considering various disease aspects, including clinical and serological features, Boolean remission provides a more holistic approach to monitoring disease activity. Achieving Boolean remission in SLE is associated with reduced organ damage and improved quality of life [16].

Ankylosing Spondylitis

In ankylosing spondylitis (AS), a comprehensive remission definition that encompasses both clinical and laboratory parameters is valuable. Boolean remission aids in assessing disease control and guiding treatment decisions in AS. Achieving Boolean remission in AS is associated with improved physical function and a reduced risk of structural damage [17].

Advantages of Boolean remission

Improved Patient Outcomes

One of the primary advantages of adopting Boolean remission criteria in the management of rheumatic diseases is the potential for improved patient outcomes. Unlike traditional remission criteria, which may focus solely on specific markers, Boolean remission comprehensively considers clinical, biochemical, and patient-reported outcomes [4]. This holistic approach ensures that not only are disease symptoms controlled, but the patient's overall well-being and quality of life are also prioritized [18].

Standardization of Treatment Protocols

Boolean remission provides a standardized and rigorous framework for assessing treatment response in rheumatic diseases [4]. By clearly defining remission based on multiple parameters, it guides clinicians in tailoring treatment strategies to achieve optimal outcomes. Standardization minimizes subjectivity and supports evidence-based decision-making, leading to more effective and consistent patient care [19].

Utility in Clinical Trials

Boolean remission criteria have gained prominence in clinical trials for rheumatic diseases due to their robustness and objectivity [20]. Researchers increasingly rely on Boolean remission as a primary endpoint, as it offers a clear and measurable target for evaluating the efficacy of new treatments.

Limitations and criticisms

Sensitivity and Specificity

One of the primary limitations of Boolean remission criteria lies in their sensitivity and specificity [21]. While these criteria provide a stringent definition of remission, they may occasionally fail to capture mild disease activity or subtle changes in patient status [22]. This can lead to missed opportunities for treatment adjustment in cases where a patient is close to achieving full remission but does not meet all criteria.

Applicability in Diverse Patient Populations

The applicability of Boolean remission criteria across diverse patient populations is another area of concern. Rheumatic diseases can manifest differently in various ethnic groups, genders, and age cohorts [23]. Boolean remission criteria may not adequately account for these variations, potentially leading to disparities in treatment goals and outcomes [19].

Comparison with Other Remission Criteria

Boolean remission criteria are just one of several sets of remission criteria used in rheumatic disease management [4]. Comparing and choosing among these criteria can be challenging, and there is ongoing debate about which set of criteria is the most appropriate for specific diseases or patient profiles [10]. This lack of consensus can create confusion for clinicians and researchers.

Boolean remission and quality of life

Patient-Reported Outcomes

Boolean remission in rheumatic diseases has a direct impact on patients' quality of life. By considering patient-reported outcomes, including pain, physical function, and overall well-being, Boolean remission criteria ensure that treatment goals align with the experiences and needs of patients [24]. Achieving Boolean remission often leads to improvements in these patient-reported outcomes, enhancing the overall quality of life [25].

Economic Impact

Boolean remission can also have a significant economic impact. Patients in remission typically require fewer healthcare resources, including medications, hospitalizations, and surgeries [26]. This reduction in healthcare utilization not only lowers healthcare costs but also allows patients to return to work and daily activities, contributing to their economic well-being [27].

Psychological Well-Being

Achieving Boolean remission can positively influence psychological well-being. Rheumatic diseases can be physically and emotionally challenging, leading to anxiety, depression, and decreased quality of life [28]. Attaining remission provides relief from pain and disability, alleviating psychological distress and enhancing patients' overall mental health [29].

Future directions

Emerging Technologies and Biomarkers

The future of Boolean remission in rheumatic diseases holds promise with the integration of emerging technologies and biomarkers [30]. Advanced imaging techniques, such as musculoskeletal ultrasound and magnetic resonance imaging, can provide detailed assessments of joint inflammation and damage [31]. Biomarkers, including genetic markers and novel serological indicators, may enhance the accuracy and specificity of Boolean remission criteria [32]. These innovations can lead to more precise disease monitoring and treatment optimization.

Personalized Medicine Approaches

The concept of personalized medicine is gaining traction in rheumatic disease management [33]. Tailoring treatment strategies to individual patient profiles, including genetic, environmental, and clinical factors, can optimize the chances of achieving Boolean remission. Advances in pharmacogenomics and the development of targeted therapies offer exciting avenues for personalized approaches [34].

Policy Implications

Boolean remission has the potential to inform healthcare policies and guidelines. Governments and healthcare organizations may consider the integration of Boolean remission criteria into healthcare quality metrics and reimbursement policies [35]. This can incentivize healthcare providers to adopt treat-to-target strategies that prioritize Boolean remission as a treatment goal, ultimately benefiting patients and the healthcare system.

## Conclusions

Boolean remission stands as a revolutionary paradigm in the management of rheumatic diseases, offering a multi-dimensional and patient-centered approach to treatment and evaluation. Shifting away from traditional, medication-centric models, this comprehensive framework encompasses clinical, biochemical, and patient-reported measures to set a rigorous standard for assessing treatment effectiveness. Essential to achieving Boolean remission are pharmacological interventions like immunomodulators and biologics, while surgical procedures, such as joint replacements, serve as valuable adjuncts in severe cases. The synergy of medicine and surgery through interdisciplinary teams further amplifies the likelihood of sustained remission, optimizing patient care on multiple fronts. With its growing importance in clinical practice, policy-making, and research, Boolean remission is poised for further refinement through emerging technologies and personalized medicine, heralding a new era of improved outcomes and enhanced quality of life for patients.
